# A case report of heterochronic presentation of a bilateral tubal pregnancy

**DOI:** 10.1002/ccr3.8072

**Published:** 2023-10-25

**Authors:** Zeinab Mansouri, Azam Tarafdari, Sepideh Azizi, Bahareh Mehdikhani, Amirali Shababi, Farzad Vaghef Davari

**Affiliations:** ^1^ Department of Obstetrics & Gynecology, Imam Khomeini Hospital complex Tehran University of Medical sciences Tehran Iran; ^2^ Shahid Akbarabadi Clinical Research Development Unit (ShACRDU) Iran University of Medical Sciences (IUMS) Tehran Iran; ^3^ Department of Radiology Iran University of Medical Sciences Tehran Iran; ^4^ School of Medicine Iran University of Medical Sciences Tehran Iran; ^5^ Tehran University of Medical Sciences‐Cancer Institute Tehran Iran

**Keywords:** bilateral, ectopic pregnancy, MTX, salpingostomy, treatment

## Abstract

**Key Clinical Message:**

This paper highlights that diagnosis and treatment of one ectopic pregnancy does not rule out the happening of a second ectopic pregnancy in the same patient concurrently, especially if the patient has rising β‐hCG and persistent symptoms.

**Abstract:**

Bilateral tubal pregnancy (BTP) is the most uncommon form of tubal ectopic pregnancy. Complications can lead to maternal morbidity and mortality. We reported a case of left tubal pregnancy and the patient underwent laparoscopic salpingostomy. During the follow‐up, the contralateral ectopic pregnancy was discovered and treated with MTX.

## 1 INTRODUCTION

Ectopic pregnancy is a common emergency but life‐threatening condition that obstetricians and gynecologists face and requires fast and careful management. Ectopic pregnancy (EP) is defined as the extra uterine implantation and development of the blastocyst. The incidence of EP has raised in developed countries in the past 30 years.^1^ The leading risk factors of EP in women are pelvic inflammatory disease, previous pelvic surgery, tubal infertility, and congenital uterine abnormalities.^2^, ^3^ While unilateral tubal pregnancy accounts for 90% of all EPs, bilateral tubal pregnancy (BTP) is considered the most uncommon form.^4^ It is difficult to estimate the accurate frequency which is based on case reports; however, the highest reported incidence was 1 in 200,000 pregnancies.^5^ This demonstrates 1/750–1500 of all EPs.^6^ Complications such as severe bleeding and hypovolemic shock associated with maternal morbidity and mortality are the results of misdiagnosis or delayed diagnosis of EP.^2^, ^3^ In this case, we report a patient with bilateral tubal pregnancy, and the presentations were not at the same time which makes it more life‐threatening and harder to diagnose.

## 2 CASE PRESENTATION

A 22‐year‐old woman, gravid 2, abortion 1 (spontaneous, gestational age(GA) <10 weeks) with a negative past medical history of sexually transmitted disease or infertility presented to the Emergency Department complaining of 2 weeks of delayed menses, spastic abdominal pain and spotting. She was wrongly administered one tablet of Letrozole 2.5 mg orally for 10 days and also injected one dose of subcutaneous human chorionic gonadotropin (HCG) previously. Her vital signs were stable. The abdominal examination revealed tenderness on LLQ with absent bowel sounds. Pelvic bimanual examination revealed a left adnexal fullness. Laboratory data revealed a serum Beta HCG level of 2700 mIU/mL and mild anemia (Hemoglobin level = 9 mg/dL).

The ultrasound evaluation showed a complex mass (50 × 70 mm) suggestive of ectopic pregnancy and hematoma in the left adnexa with moderate free abdominopelvic fluid. The right tube was intact. There was no evidence of an intrauterine pregnancy at that time.

The patient underwent laparoscopic surgery; 1 L of hemoperitoneum was drained. There was an unruptured left‐sided ampullary ectopic pregnancy measuring ∼3 × 3 cm; the products of conception were removed via salpingostomy, suction, and irrigation. The right fallopian tube was normal. We inspected the abdomen and pelvis carefully, but we did not find any other abnormal findings.

On the first postoperative day, the serum Beta HCG level was 1300 mIU/mL (50% drops). On the next week's follow‐up, the serum Beta HCG level elevated to 1600 mIU/mL. The ultrasonography reported a round hypoechoic right adnexal mass (15 × 12 mm) which was highly suggestive of ectopic pregnancy. The left adnexa was unremarkable. The uterine cavity was empty. The patient with the diagnosis of bilateral ectopic pregnancy was a candidate for medical therapy. Methotrexate 1 mg/kg intramuscular was injected. The serum Beta HCG level was reported 2300 mIU/mL on both the 4th and 7th days after medical treatment.

The patient was re‐evaluated via ultrasonography, which reported the increased size of the right adnexal mass due to the complex of ectopic pregnancy and hematoma. Because the serum Beta HCG level was not reduced by at least 15% in comparison to the 4th and 7th day of the treatment, she received a second dose of methotrexate. After the second methotrexate dosage, the serum Beta HCG level was reduced to zero. During the 1‐month follow‐up period, the patient had no complications.

## 3 DISCUSSION

Ectopic pregnancy is a common emergency but life‐threatening condition that obstetricians and gynecologists face and requires fast and careful management. In the past 30 years, the incidence of EP has raised in developed countries.^1^ Approximately 1%–2% of all pregnancies are ectopic pregnancies and over 98% of implantations occur in the fallopian tube.^7^ The leading risk factors of EP in women are pelvic inflammatory disease, previous pelvic surgery, tubal infertility, and congenital uterine abnormalities.^2^, ^3^

Bilateral tubal pregnancy (BTP) is considered an extremely rare form of extra‐uterine pregnancy.^4^ It is difficult to estimate the accurate frequency which is based on case reports; however, the highest reported incidence was 1 in 200,000 pregnancies.^5^ This demonstrates 1/750–1500 of all EPs.^6^ BTP is commonly associated with infertility treatment. Multiple ovulations either spontaneously or with ovulation induction have elevated the risk of bilateral EP.^8^ In addition, the incidence has increased due to the high rates of endosalpinx damages following sexually transmitted infections, tubal sterilizations, assisted reproductive technologies, tobacco smoking, polygamy, and more precise methods for early detection of ectopic pregnancy. The highest risk factor for ectopic pregnancy is previous fallopian tube damage.^9^, ^10^, ^11^

Complications such as severe bleeding and hypovolemic shock associated with maternal morbidity and mortality are the results of misdiagnosis or delayed diagnosis of EP.^2^, ^3^ In this case, we report a patient with bilateral tubal pregnancy, and the presentations were not at the same time which makes it more life‐threatening and harder to diagnose.

In cases of unilateral EP, early diagnosis can be successfully achieved with the use of transvaginal ultrasonography (TVUS) and the accessibility of β‐hcg kits. Transvaginal ultrasonography has a high sensitivity and specificity for detecting EP and Color Doppler sonography increases the rate of transvaginal ultrasound for early detection of small ectopic masses, preoperatively.^7^, ^12^ However, ultrasound has a weak role in the diagnosis of bilateral EP, and almost all cases are diagnosed intraoperatively, because even the most expert sonographers may encounter a handful in their life career.^13^ The literature review has shown it as an operative diagnosis, except for only a few reports. Commonly, clinicians fail to recognize the diagnosis during the surgery as seen in our patient.^12^

In our case, we did not visualize any abnormal findings on the contralateral tube during the laparoscopic surgery. During the post‐operative follow‐up due to the rise of β‐hcg level and evaluating other possibilities, we found out about the other ectopic pregnancy in the contralateral tube. We concluded that during laparoscopic surgery, the other tubal pregnancy was too small to be seen then.

BTP treatment is controversial in most clinical guidelines for the management of EP, because of the rare cases of BTP.^14^ Treatment options for EP management are surgery, medical therapy, and expectant management. Systemic MTX therapy is considered a cost‐effective choice rather than laparoscopy for patients who have stable hemodynamics. Periodic β‐hCG measurements are useful to diagnose EP and to assess the efficacy of MTX.^15^

Clinicians should know about various therapeutical alternatives. Like unilateral EP, choices depend on the patient's condition, the extent of damage to the fallopian tubes, the desire to preserve fertility, the size and location of the EP mass, and the β‐hCG level.^16^

Therapeutic options for spontaneous BTP cases are essentially similar to unilateral EP. The type of surgical procedure may be different between spontaneous BTP and those following ART. In spontaneous BTP, if the tube appears benign, the procedure is to perform a salpingostomy. This is the only successful pregnancy in the series.^17^

Yao and Tolandi^18^ compared fertility rates between salpingotomy versus salpingectomy, showing that both approaches were the same. Femke et al.^19^ demonstrated a similar cumulative rate of natural pregnancy among mentioned approaches.

Conservative management using methotrexate (MTX) does not have satisfactory therapeutic effect due to the high hCG level (the hCG concentration level for MTX treatment was <5000 IU/L),^20^ salpingotomy should be considered if the patient has a strong desire to save fertility. However, patients should be aware of the possibility of trophoblast tissue remnant and persistent trophoblast, complementary treatment with systemic MTX, and EP recurrence.^19^, ^21^

In our case, first, we did a laparoscopic salpingostomy and after the diagnosis of BTP, we treated the patient with MTX therapy which needed a second dosage.

Recent reports demonstrated delayed diagnosis of contralateral tubal pregnancy days to weeks after the first surgery in BTP, so clinicians should always keep in mind such an alternative, particularly, in patients for whom multiple embryo transfers have been performed. Bilateral fallopian tubes should always be checked, and inconsistent β‐hCG values may delay accurate diagnosis.^15^ In our case, we were able to diagnose it after the surgery and the rise of β‐hCG level to search for other possible locations of pregnancy.

Surgical procedures vary from salpingectomy for one tube and linear salpingostomy for a contralateral tube to bilateral salpingostomy or bilateral salpingectomy.^21^ If present, laparoscopy may be the best choice for diagnosis and management of BTP^22^ because the diagnosis can be easily missed even at laparoscopy so a high index of suspicion should be kept.^22^ But in our case, as we inspected the contralateral tube thoroughly we did not observe any abnormal findings due to the possibility of a small ectopic pregnancy in the right tube.

In hemodynamically unstable patients, laparotomy is the choice treatment and is equally impressive. However, it should be noted that serial β‐hCG monitoring should be performed because there is a possibility of ongoing ectopic pregnancy, especially if a conservative surgery such as salpingostomy or tubal milking has been chosen.^16^ There are reports of intrauterine pregnancy after conservative surgical management of BTP,^5^ but the reality is that these patients are at high risk of recurrent ectopic pregnancy subsequently.^16^

The unusual part of this case is the chronology of the bilateral ectopic pregnancy. Most reported bilateral ectopic pregnancies occurred and developed simultaneously, so the diagnoses were made at the same time on both sides. In this case, even the mindful exploration of the contralateral fallopian tube did not show any signs of a developing ectopic pregnancy. At the first exploration, the contralateral implanted embryo was too underdeveloped to be seen by laparoscopy, which contributed to the consequently delayed diagnosis of bilateral ectopic pregnancy. In this paper, we present an unusual case of bilateral ectopic pregnancy, in which there has been a substantial delay of 2 weeks between the diagnoses of both sides of the ectopic pregnancies.

## 4 CONCLUSION

To begin with first of all, we should consider have this diagnosis in mind. Evaluation of both adnexa by TVUS is equally important. A watchful inspection of the abdomen and pelvis should always be performed during the surgery for an ectopic pregnancy, particularly the contralateral fallopian tube even when unilateral EP is diagnosed preoperatively. In some cases, contralateral pregnancy was shown days to weeks after the first surgery. This paper accentuates that diagnosis and treatment of one ectopic pregnancy does not rule out the happening of a second ectopic pregnancy in the same patient concurrently at the same time, especially if the patient has rising β‐hCG and persistent symptoms.

## AUTHOR CONTRIBUTIONS

**Zeinab Mansouri:** Conceptualization; data curation; formal analysis; funding acquisition; investigation; methodology; project administration; resources; software; supervision; validation; visualization; writing – original draft; writing – review and editing. **Azam Tarafdari:** Data curation; investigation; project administration; resources; supervision; writing – review and editing. **Sepideh Azizi:** Data curation; formal analysis; methodology; resources; software; validation; writing – original draft; writing – review and editing. **Bahareh Mehdikhani:** Data curation; methodology; resources; software; supervision; validation; visualization; writing – original draft; writing – review and editing. **Amirali Shababi:** Software; writing – original draft. **Farzad Vaghef Davari:** Investigation; project administration.

## CONFLICT OF INTEREST STATEMENT

The authors report no conflict of interest.

## CONSENT

Written informed consent was obtained from the patient to publish this report in accordance with the journal's patient consent policy.

## Data Availability

The data that support the findings of this study will be available on request of the Editor in chief.
